# Towards parsimony in habit measurement: Testing the convergent and predictive validity of an automaticity subscale of the Self-Report Habit Index

**DOI:** 10.1186/1479-5868-9-102

**Published:** 2012-08-30

**Authors:** Benjamin Gardner, Charles Abraham, Phillippa Lally, Gert-Jan de Bruijn

**Affiliations:** 1 Health Behaviour Research Centre, Department of Epidemiology & Public Health, University College London, Gower Street, London, WC1E 6BT, UK; 2 Peninsula College of Medicine & Dentistry, University of Exeter, St. Luke’s Campus, Heavitree Road, Exeter, EX1 2LU, UK; 3 Amsterdam School of Communication Research, University of Amsterdam, Kloveniersburgwal 48, 1012 CX, Amsterdam, The Netherlands

**Keywords:** Habit, Automaticity, Self-report, Measurement, Energy‐balance related behaviours

## Abstract

**Background:**

The twelve-item Self-Report Habit Index (SRHI) is the most popular measure of energy-balance related habits. This measure characterises habit by automatic activation, behavioural frequency, and relevance to self-identity. Previous empirical research suggests that the SRHI may be abbreviated with no losses in reliability or predictive utility. Drawing on recent theorising suggesting that automaticity is the ‘active ingredient’ of habit-behaviour relationships, we tested whether an automaticity-specific SRHI subscale could capture habit-based behaviour patterns in self-report data.

**Methods:**

A content validity task was undertaken to identify a subset of automaticity indicators within the SRHI. The reliability, convergent validity and predictive validity of the automaticity item subset was subsequently tested in secondary analyses of all previous SRHI applications, identified via systematic review, and in primary analyses of four raw datasets relating to energy‐balance relevant behaviours (inactive travel, active travel, snacking, and alcohol consumption).

**Results:**

A four-item automaticity subscale (the ‘Self-Report Behavioural Automaticity Index’; ‘SRBAI’) was found to be reliable and sensitive to two hypothesised effects of habit on behaviour: a habit-behaviour correlation, and a moderating effect of habit on the intention-behaviour relationship.

**Conclusion:**

The SRBAI offers a parsimonious measure that adequately captures habitual behaviour patterns. The SRBAI may be of particular utility in predicting future behaviour and in studies tracking habit formation or disruption.

## Background

Many energy-balance related behaviours (EBRBs; e.g., active travel, unhealthy snacking) are performed habitually, with little forethought [1]. Habits are defined as behavioural patterns, based on learned context-behaviour associations, that are elicited automatically upon encountering associated contexts [2]. Habits are acquired through context-dependent repetition [3], and, once formed, are hypothesised to have two effects on behaviour. First, where associated contexts are consistently encountered and remain stable, habit strength will correlate with behavioural frequency. Second, habit will override motivational determinants of behaviour so that, as habit strengthens, the relationship between deliberative intentions and behaviour will weaken. Subsequently, where habits and intentions conflict, behaviour will tend to proceed in line with habit and not intention [4,5]. These hypotheses have been empirically well-supported in EBRB determinant studies [4].

There is growing recognition of the importance of habit in EBRB change. Motivation-based interventions may be insufficient to break established dietary or sedentary habits, because people tend to behave in line with their habits even when motivated not to do so [6]. Effective behaviour change may depend on disrupting the cue-response relationships that support habitual EBRBs. Conversely, establishing habits for health-promoting EBRBs will facilitate behavioural maintenance, by increasing the likelihood of behaviour persisting even where motivation diminishes [7,8]. Recent work has sought to model the habit development process [3,9,10], and EBRB interventions explicitly based on habit formation principles are being trialled [11].

Assessing the extent to which EBRBs are habitual requires a practical, reliable and conceptually robust habit measure. The most popular habit measure in the EBRB domain is the Self-Report Habit Index (SRHI; [4,12]). The SRHI comprises twelve items reflecting on three proposed characteristics of habit: automaticity (e.g. [‘Behaviour X is something…’] ‘…*I do without thinking*’), frequency (‘…*I do frequently*’), and relevance to self-identity (‘…*that’s typically “me”’*). The SRHI has been found to detect the habit-behaviour association and moderation of the intention-behaviour relationship in EBRB domains [4].

Recent findings have, however, questioned the parsimony of the SRHI [4,13-15]. Various SRHI subscales have been used with no apparent losses in reliability [16-18], suggesting that some items may be redundant. The SRHI may burden participants unnecessarily, which may be especially problematic in EBRB research, in instances where a multitude of determinants are proposed [19], multiple habits are measured (e.g. soft drink consumption and TV viewing [20]), or, in longitudinal research, habits are assessed at several timepoints [11]. For example, in one weight-loss intervention trial, participants completed the 12 SRHI items in relation to 14 behaviours over three timepoints (504 items in total [11]). Unsurprisingly, dissatisfaction was expressed with questionnaire length. Such burden can lead to unreliable or incomplete responses, or withdrawal from the study [21]. Development of a standardised SRHI subscale may allow more ‘participant-friendly’ assessment of energy-balance related habits.

The conceptual basis of the SRHI has also been questioned. Strong reliability coefficients and a single factor structure have been interpreted as support for a conceptualisation of habit based on three identifiable components: automaticity, behavioural frequency and identity [22]. However, higher numbers of items biases alpha coefficients towards higher values, and factor analyses may be insensitive to potentially distinct factors on which only one item loads (e.g. identity). A more robust analysis, in which the SRHI was supplemented by additional self-identity items, found that the single SRHI identity item loaded on to a separate factor from all other SRHI items [14], suggesting that identity-relevance is not a necessary component of a habit.

Moreover, the incorporation of behaviour frequency indicators in the SRHI is problematic when estimating the relationship between habit and behaviour frequency [4,14,15]. Established habits can only be distinguished from frequent intentional behaviours by automatic activation [1,23]. Commentators have thus proposed that the effects of habit on action can be attributed to automaticity [15,24], and that it is because habits are automatically elicited that habitual behaviour persists in associated contexts, and deliberative tendencies are overridden [5,25]. According to this viewpoint, repeated performance is an antecedent (and consequence) of automaticity [3,15,23], and so the contribution of past behaviour to habit should be reflected by the extent to which behaviours are automatically activated. While behavioural frequency items may be needed to distinguish habit from automatic actions which do not develop through repeated performance, this distinction is rarely of interest in EBRB prediction and habit formation or disruption studies. An automaticity measure may therefore adequately capture habit in these settings. Frequency items are also problematic from a practical perspective, because they can incorporate unidentified stable influences on behaviour [26], and so can inflate habit-behaviour associations [4]. It has been suggested that frequency indicators may not be needed to detect a moderating effect of habit-related automaticity on the intention-behaviour relation [4]. Gardner et al. [4] called for an “SRHI subscale which removes frequency and so may permit a truer estimate of the relationship between cue-response association strength and behavioural performance” (p185).

### The present study

This paper describes work to identify and test a SRHI subscale based on behavioural automaticity. Identification of an SRHI shortform would have conceptual and practical benefits for EBRB prediction and habit formation studies. Although automaticity-specific SRHI subscales have been used to study EBRBs [3,16-18], no attempt has been made to systematically identify automaticity indicators within the SRHI, and so there has been disagreement about which items best capture automaticity.

We used content validity procedures to identify SRHI items consensually agreed by a panel of researchers to capture automaticity. The convergent validity and predictive utility of the resultant automaticity scale was tested using data from two sources. First, corresponding authors of published SRHI studies, identified via systematic review, were asked to re-analyse their findings using the automaticity subset, and these data were meta-analysed where possible. To maximise data availability, data from all behavioural domains were eligible for analysis. Second, two new primary datasets were collected. Previous SRHI studies have been criticised for neglecting contexts in which habit and intention measures conflict [4], and potential contextual cues to habitual action [15]. To assess the utility of the automaticity subset in these settings, one new dataset measured habits (for unhealthy snacking) alongside counterhabitual intentions (to avoid eating unhealthy snacks), and one dataset used habit items worded to include a potential cue (‘drinking alcohol *with the evening meal*’). Availability of primary datasets in raw form enabled comparisons between the automaticity index and a composite of SRHI items removed from the automaticity subset, which we did not deem feasible to request from authors of published studies. A further two datasets, which formed the basis of previously published work [16], were also available to us in raw form and permitted comparisons with an additional habit measure (the transport-specific ‘Response-Frequency Habit Measure’ [27]). Together, the four primary datasets covered both sides of the energy balance equation: energy expenditure (inactive and active commuting) and intake (snacking, alcohol consumption [28]).

In all analyses, the automaticity index (which we term the ‘Self-Report Behavioural Automaticity Index’; SRBAI) was assessed against the criteria by which the SRHI has been tested and become established: reliability, convergent validity, and predictive validity. We hypothesised that:

*Hypothesis 1.* (a) The SRHI and SRBAI (and RFM) will be intercorrelated, and (b) will each correlate with behaviour. However, (because of the removal of items which assess frequency and identity) (c) the automaticity-specific SRBAI will be less strongly correlated with behaviour than will the SRHI, or (d) a scale comprised of SRHI items removed from the SRBAI.

*Hypothesis 2.* (a) The SRBAI and SRHI will each moderate the relationship between intentions and behaviour, such that where habit is strong, intentions will have a weaker effect on behaviour, and vice versa. Assuming that the moderating effect of habit is attributable to automaticity, then, due to the removal of strong automaticity indicators, (b) a composite of SRHI items omitted from the automaticity subset will fail to detect moderation of the intention-behaviour relationship.

Support for Hypotheses 1a, 1b and 2a would show that the SRBAI can capture habit-behaviour effects to the same extent as the SRHI. Support for Hypotheses 1c, 1d and 2b would suggest that the SRBAI excludes items that may exaggerate true habit-behaviour associations or obscure the expected habit x intention interaction.

Hypotheses 1a, 1b, 1c and 2a were assessed using secondary data and the four primary datasets. Hypotheses 1d and 2b were assessed using the four primary datasets only.

## Methods

### Identification of SRHI automaticity items

We used Discriminant Content Validation [29], which permits statistical analyses of the consistency of raters’ judgements of face validity, to systematically extract automaticity items from the SRHI. Judges were seven active social or health psychology researchers (not the present authors), with expertise in social cognition but little or no experience of conducting habit-related research. Judges were asked to estimate whether each of the twelve SRHI items mapped on to literature-based definitions of automaticity, frequency, and self-identity (yes vs no), and to rate their confidence for each judgement on an 11-point scale (0 = completely uncertain; 10 = completely certain). Each judgement (yes [+1] vs no [−1]) was multiplied by its confidence rating, producing inter-rater scores ranging from −10 (completely certain that item does not match construct), through 0 (complete uncertainty), to +10 (completely certain item matches construct). One-sample t-tests with a test value of zero independently classified each item in relation to each of the three constructs.

Seven items (items 2, 3, 5, 7, 8, 9, and 10 of the SRHI) were judged to measure automaticity only. Items 2 ([‘Behaviour X is something…’] *‘I do automatically’*), 3 (*‘I do without having to consciously remember’*), 5 (*‘I do without thinking’*) and 8 (*‘I start doing before I realize I’m doing it’*) were most confidently and consistently judged to capture automaticity (*t*s > 45.00, *p*s < .001). To maximise parsimony and content validity, these four items were selected to represent automaticity in subsequent analyses, on the basis that each judge was at least 90% certain that each item represented automaticity (minimum mean inter-rater score = 9.57, maximum SD = 0.54). The four-item composite is hereafter referred to as the ‘Self-Report Behavioural Automaticity Index’ (SRBAI).

Details of the content validation task are available from the first author.

### Data collection

#### Secondary datasets: Systematic search strategy and results

Five psychology and health databases (PsycInfo, Medline, Embase, Web of Knowledge, Scopus) were searched on 20th April 2011. In each, Verplanken and Orbell’s [12] SRHI paper was located and citing articles subsequently identified. No date limits were set. Verplanken & Orbell’s paper, and three then-in-press papers [30-32], were added.

Papers were eligible for inclusion if they were (a) written in English, (b) published full-text in peer-reviewed journals, and (c) reported findings from a primary dataset which included (d) the 12-item SRHI as (e) a measure of habit in relation to a behaviour. Papers focusing on habitual thought or emotion (e.g. [33]) were excluded.

Papers were retained only when they reported (a) the reliability of the SRHI (Cronbach’s alpha), (b) the correlation between the SRHI and a matched (habit-consistent) or directly opposed (counterhabitual) behaviour (Pearson’s r), and/or (c) a test of the moderating effect of the SRHI on the relationship between intention and a habit-consistent or counterhabitual behaviour (using moderated multiple regression [MMR], whereby behaviour was modelled on habit, intention, and a means-centred ‘habit x intention’ interaction term [34]).

316 papers were identified, of which 135 were duplicates (see Additional file 1: Figure S1 for search and screening flow chart). Title and abstract screening excluded 7 papers, and full-text screening excluded a further 85 papers. 47 papers, based on 49 unique datasets, were retained. BG screened all papers. PL independently screened 20% of papers. 100% agreement was recorded on selection criteria and data extraction.

Authors of eligible papers were contacted by email, and asked to generate an SRHI-SRBAI correlation coefficient and rerun, using the SRBAI, as many of the three analyses (reliability, correlation with behaviour, moderation of intention-behaviour relation) as had been reported in the published paper. Tailored SPSS syntax templates were sent to aid re-analysis. Authors were invited to alternatively send clearly labelled SPSS datasets to allow us to conduct reanalyses. Authors were also asked to indicate where datasets had been used for multiple publications.

Authors were instructed to assess reliability using Cronbach’s alpha, to generate bivariate Pearson’s r correlation coefficients, and to use MMR to investigate moderation, using a composite of the four SRBAI items (‘[Behaviour X is something…]*’ ‘I do automatically’, ‘I do without having to consciously remember’, ‘I do without thinking’, ‘I start doing before I realise I’m doing it’*). We requested that MMR re-analyses control for the same variables as in the published paper and that, regardless of statistical significance of the habit x intention interaction term, simple slope analysis be undertaken to model intention effects at differing habit levels.

Where habit or behaviour was measured at multiple timepoints, we requested habit data from the earliest timepoint, and behaviour data from the earliest follow-up. Where papers reported intervention evaluations, we invited either (a) baseline data only for all participants combined, or (b) baseline habit and follow-up behaviour data for a no-treatment control group. Where a single dataset contained multiple habit measures, relevant data were obtained for all measures.

Twenty-seven authors were contacted. 21 provided all requested information, of whom 14 sent re-analyses and 7 provided raw datasets. Three authors did not provide sufficient information for all possible re-analyses, and three did not respond, thereby excluding 7 papers. Two datasets were excluded because the raw data were entered into the primary analyses reported below ([16], Studies 1 and 2).

The final dataset included 34 unique datasets (from 39 papers), generating 45 tests of reliability and SRHI-SRBAI correlations. Habit-behaviour correlations were available from 24 datasets (allowing 28 tests), and moderation could be tested in 5 datasets (7 tests).

#### Primary datasets

Details of the two previously published datasets, which used prospective designs and related to car (Dataset 1; *N* = 105) and bicycle commuting (Dataset 2; *N* = 102) respectively, are available elsewhere [16], though the RFM was excluded from the published report. In Datasets 1 and 2, participants completed ten RFM items, which were preceded by instructions asking participants to indicate, as quickly as possible without much deliberation, whether they would use a car, bus, train or any other transport mode in each of ten scenarios (e.g. ‘visiting a friend’, ‘taking a trip on a nice day’ [35]). Each item was presented for a maximum of 10 seconds, and any-key responses prompted presentation of the next item. Participants completed 5 practice trials. RFM scores represented the summed frequency with which the car (or bicycle) was chosen. SRHI-based habit indices, including a composite scale of SRHI items removed from the SRBAI (i.e. SRHI items 1, 4, 6, 7, 9, 10, 11, and 12 [e.g. *‘Behaviour X is something I have no need to think about doing’*[12]; hereafter, the ‘non-SRBAI’), were reliable in both datasets (Dataset 1: minimum α = .92; Dataset 2: minimum α = .91).

The two new datasets were collected via online questionnaires. All measures were self-reported, and unless otherwise specified, responses ranged from 1 (strongly agree) to 7 (strongly agree). A priori power analysis for a medium-effects regression model with three predictors indicated that N = 76 was sufficient for power at .80 where p < .05.

Dataset 3 (snacking) used a prospective design, whereby habit and intention were measured at Time 1, and behaviour was measured via email one week later (T2). Participants were recruited via an email sent to participant pool lists, and recipients were encouraged to forward the email to others to create a ‘snowball’ effect [36]. 188 UK non-diabetic adults with no eating disorders completed all study items (49 males, 138 females, 1 unspecified; age range 18–76, M = 30.94, SD = 11.77). *Behaviour* was measured as the frequency with which each of five high-calorie snacks (crisps, chocolate, cakes, sweets, biscuits) were eaten over the previous week (*‘0 times’*[1] – *‘10 or more times’*[7]), as summed to provide an index (range 1–35; M = 10.32, SD = 2.89). *Intention* measures related to *avoiding* eating high-calorie snacks (e.g. ‘I will try to avoid eating high-calorie snacks over the next 7 days’; 3 items, α = .92; M = 4.06, SD = 1.81), and *habit* items related to ‘eating high-calorie snacks’ (e.g. ‘…is something I do automatically’). Habit indices were reliable (minimum α = .81), and mean scores suggested moderate snacking habits (SRHI: M = 3.50, SD = 1.19; SRBAI: M = 3.39, SD = 1.55; ‘non-SRBAI’: M = 3.55, SD = 1.13).

Dataset 4 (alcohol consumption) used a cross-sectional design. 204 members of a UK-based health research panel, recruited via an email advertisement, completed all study items (50 males, 150 females, 4 unspecified; age data not recorded due to researcher error). (Past) *behaviour* was calculated as a percentage, using a) number of evening meals with which at least one alcoholic drink was consumed, and b) number of evening meals eaten, over the preceding week (i.e. [a/b x 100]; M = 27.57, SD = 28.05). *Intention* was measured using two items (e.g. ‘I intend to drink an alcoholic drink with my evening meal on most days over the next week’; α = .94; M = 2.11, SD = 1.80). *Habit* items related to ‘drinking an alcoholic drink with my evening meal’ (e.g. ‘…is something I do without having to consciously remember’; minimum α = .92). Mean scores indicated typically weak habits (SRHI: M = 2.14, SD = 1.41; SRBAI: M = 1.90, SD = 1.35; ‘non-SRBAI’: M = 2.26, SD = 1.48).

Further details of all primary datasets are available on request from the first author.

### Analysis strategy

Data were analysed using procedures and techniques commonly used to quantify the contribution of the SRHI to prediction of behaviour (e.g. [4]).

#### Analysis of secondary datasets

Correlation coefficients were entered into meta-analysis to generate weighted summary effects for comparison. Within-dataset and meta-analysed SRBAI-behaviour and SRHI-behaviour correlations were compared statistically by the present authors, following Meng, Rosenthal and Rubin’s guidelines [37]. Moderation effects, as partial correlations, were not meta-analysed because of variation across studies in the variables controlled within MMR models. Instead, a vote-count procedure was employed. Where sample sizes were inconsistent across effects in a single dataset (e.g. larger N available for reliability than for correlations; 16 datasets), the smallest N was used for all effects generated from that dataset.

Fixed-effect meta-analysis of correlations was undertaken using Comprehensive Meta-Analysis software [38]. Sample-weighted average effect sizes (*r*_*+*_) were calculated using Fisher’s *Z* transformations, and 95% confidence intervals were generated. Negative correlations between habit and counterhabitual behaviour were reversed prior to weighting. A priori power analysis, conducted using conservative estimates for unknown parameters, indicated that, for 27 tests where average within-study *N* = 50 and one-tailed *p* < .05, power to detect a small effect (*r*_*+*_ = .10) was 0.84 [39]. Bivariate Pearson’s r correlations of .10, .30, and .50 were interpreted as small, medium and large effects respectively [40].

Three datasets yielded multiple and conceptually independent habit-behaviour correlations (e.g. TV viewing habits and behaviour, soft drink consumption habits and behaviour). All such effects were retained, but sample sizes were divided by the number of relevant habit-behaviour correlations, and rounded downwards where this did not produce an integer (e.g. 538 / 3 = 179.33 ≈ 179 [30,31,41]). While this violates the independence assumption of meta-analysis, we do not view this as problematic, because analysis was undertaken to compare habit measures, not to generate reliable effect size estimates.

#### Analysis of primary datasets

Cronbach’s alpha coefficients were calculated to test the reliability of the SRHI, SRBAI, and ‘non-SRBAI’ indices. Pearson’s r correlation coefficients were generated for relationships between the habit indices and behaviour, and differences in the magnitude of habit-behaviour correlation coefficients were evaluated statistically [37].

MMR was used to test for moderation. Behaviour was modelled on habit, intention, and a ‘habit x intention’ interaction term (i.e. the multiplicative product of means-centred habit and intention variables). Significant interaction terms denote moderation, and were deconstructed using simple slope analysis to plot intention-behaviour effects at one SD below the mean (weak habit), at the mean (moderate habit), and one SD above the mean of the habit variable (strong habit [34]). Multiple models were run to compute effects for each habit index (SRHI, SRBAI, ‘non-SRBAI’).

## Results

### Secondary datasets

#### Reliability

Of 45 reliability assessments of the SRBAI, 23 found α within the range .90-.97, 17 found an alpha between .80-.89, four an alpha between .70-.79, and one alpha equalled .68 (see Additional file 2: Table S1 for study characteristics). The SRBAI thus appeared largely reliable.

#### Correlations of SRHI, SRBAI, and behaviour

The SRHI and SRBAI correlated at *r*_*+*_ = .92 (95% CI: .91, .92, *p* < .001; range ± .79-.97; *k* = 45, *N* = 11,257; Table 1), supporting Hypothesis 1a. Of 28 habit-behaviour correlation tests, 21 found the SRBAI-behaviour correlation to be lower than the SRHI-behaviour correlation (*p* < .05), and in 7 there was no difference. Across tests, the weighted SRBAI-behaviour correlation (*r*_*+*_ = .41; 95% CI: .39, .43, p < .001) was significantly lower than the SRHI-behaviour correlation (*r*_*+*_ = .47; 95% CI: .45, .48, *p* < .001; *Z* = 14.31, *p* < .001; *k* = 28, *N* = 8,492). Hypotheses 1b and 1c were mostly supported.

**Table 1:** ***Secondary datasets: *Meta-analysis of SRHI-SRBAI and habit-behaviour correlation coefficients**

**k**	**N**	**SRHI-SRBAI *r *(95% CI)**	**Habit-behaviour**		
			**SRHI**	**SRBAI**	**Z**
			**r (95% CI)**	**r (95% CI)**	
45	11,257	.92***			
		(.91, .92)			
28	8,492		.47***	.41***	14.31***
			(.45, .48)	(.39, .43)	

***p < .001. k = number of datasets, N = total number of participants across datasets, Z = test for difference between SRHI-behaviour and SRBAI-behaviour correlation coefficients (see [37]).

#### Moderation tests

Of 7 tests of the moderating impact of habit on the intention-behaviour relation, the SRHI and SRBAI yielded similar effects in 5 tests: in four tests, both measures found moderation, and in one test, neither measure found moderation (Table 2). In one test, the SRHI found a moderation effect (*p* < .04) but the SRBAI did not (*p* = .13). In another test, the SRBAI found a tendency towards moderation that approached statistical significance (*p* = .052) but the SRHI did not (*p* = .11). The latter test generated an unexpected ‘effect’ whereby the impact of intentions on behaviour increased as habit strengthened; a similar pattern of intention-behaviour relations at varying habit levels was also observed here using the SRHI. Hypothesis 2a thus received mixed support.

**Table 2:** ***Secondary datasets: *SRHI vs SRBAI as moderator of the intention-behaviour relationship**

**Reference**	**N**	**Habit**	**Intention**	**Covariates in regression model**	**SRHI**				**SRBAI**			
					**Significance of moderation effect (p)**	**Intention-behavior β (p)**			**Significance of moderation effect (p)**	**Intention-behavior β (p)**		
						**Weak or no habit**	**Moderate habit**	**Strong habit**		**Weak or no habit**	**Moderate habit**	**Strong habit**
De Bruijn [41] / *De Bruijn & Gardner *[30]*/ De Bruijn & Rhodes *[31]	538	Eating at least 2 pieces of fruit per day	To eat two pieces of fruit per day	I, H, PBC, A, SN	**.001**	.39(<.001)	.34(<.001)	.16(.01)	**.003**	.44(<.001)	.38(<.001)	.24(<.001)
*De Bruijn *[41] / De Bruijn & Gardner [30]/ *De Bruijn & Rhodes *[31]	538	Using a bicycle	To use a bicycle	I, H, PBC, IA, AA, SN, DG, DA	**.04**	.28(<.001)	.16(.004)	.15(.01)	**.13**	.31(<.001)	.23(<.001)	.22(<.001)
*De Bruijn *[41]* / De Bruijn & Gardner *[30] / De Bruijn & Rhodes [31]	538	Exercising for at least 20 mins per day	To engage in vigorous exercise	I, H, PBC, IA, AA, SN	**.01**	.22(.001)	.15(.007)	.06(.38)	**.04**	.27(<.001)	.22(<.001)	.15(.02)
De Bruijn, Kroeze et al. [42]	748	Watching the amount of fat in my diet	To watch the amount of fat in my diet	I, H, PBC, IA, AA, SN	**.007**	-.29(<.001)	-.19(<.001)	.07(.14)	**.007**	-.36(<.001)	-.23(<.001)	-.11(<.001)
De Bruijn, Kremers, Singh et al. [43]	317	Using a bicycle as a means of transportation	To use bicycle for transportation	OB, D, I, PBC, A, SN, H	**<.001**	.67(<.001)	.37(<.001)	.10(.11)	**<.001**	.56(<.001)	.37(<.001)	.18(.007)
Norman [44]	109	Binge-drinking	To engage in binge-drinking	I, H	**.11**	.28(.01)	.42(.001)	.57(<.001)	**.052**	.35(.001)	.52(<.001)	.68(<.001)
Rhodes, De Bruijn & Matheson [45]	153	Leisure time active sport or vigorous PA	To engage in PA (x) times per week	I, H, PBC, AA, IA, SN, IS, IxIS	**.35**	.42(.03)	.34(.001)	.78(<.001)	**.40**	.52(.005)	.38(<.001)	.48(.01)

PA = Physical activity. Covariates: I = Intention, H = Habit (SRHI/SRBAI), PBC = Perceived behavioral control, A = Attitude, IA = Instrumental attitude, AA = Affective attitude, SN = Subjective norm, IS = Intention stability, IxIS = Intention x intention stability, D = (various) demographics, OB = engagement in (various) other behaviors. DG = Demographic: Gender. DA = Demographic: Age. Italicised references indicate papers based on same data but in which the focal moderation test was not reported.

### Primary datasets

#### Correlations of habit indices and behaviour

In all four datasets, the SRBAI, SRHI and non-SRBAI indices were strongly intercorrelated (SRHI-SRBAI *r*s ≥ .90), and correlated with behaviour (*r*s ≥ .42; Table 3; see also Additional file 3: Table S2a and Additional file 4: Table S2b for full descriptive and intercorrelations). In Datasets 1 and 2, the SRBAI, SRHI and non-SRBAI also correlated strongly with the RFM (*r*s ≥ .49). Hypotheses 1a and 1b were thus supported. The SRBAI-behaviour correlation was weaker than the SRHI-behaviour correlation (*p*s < .001) in Datasets 1, 3 and 4 but not Dataset 2 (p = 1.0), and weaker than the ‘non-SRBAI’-behaviour correlation in Datasets 3 and 4 (p ≥ .04), but not Datasets 1 or 2 (minimum p = .16). There was no difference between SRHI-behaviour and ‘non-SRBAI’-behaviour correlations in any of the datasets (maximum *Z* = 1.57, minimum *p* = .06). There was mixed support for Hypotheses 1c and 1d.

**Table 3:** ***Primary datasets: *Habit indices as correlates of behaviour and moderators of intention-behaviour relationship in four primary datasets**

**Source**	**N†**	**Behaviour**	**Habit**	**Intention**	**Habit index (α)**	**Correlations††**			**Moderation of intention-behaviour relationship**				
						**SRHI-SRBAI**	**Habit-RFM**	**Habit-behaviour**	**Model R**^2^†††	**Significance of moderation effect†††† (p)**	**Intention-behaviour β**		
											**Weak or no habit**	**Moderate habit**	**Strong habit**
Dataset 1: ( [16], Study 1)	105	Inactive (car) commuting	“Using a car to commute to campus”	“To use a car to commute to campus on most days”	SRHI (.95)	.94	.52	.82^a^	.75	.001	.54***	.27*	.01
					SRBAI (.92)		.52	.76^b^	.75	<.001	.69***	.41***	.12
					Non-SRBAI (.91)		.49	.81^a^	.73	.01	.57***	.37**	.16
Dataset 2: ( [16], Study 2)	102	Active (bicycle) commuting	“Using a bicycle to commute to campus”	“To use a bicycle to commute to campus on most days”	SRHI (.95)	.97	.67	.86 ^a^	.77	.04	.16	.02	-.12
					SRBAI (.93)		.65	.86 ^a^	.77	.04	.21*	.08	-.05
					Non-SRBAI (.91)		.67	.84 ^a^	.74	.04	.26**	.12	-.02
Dataset 3: *New dataset*	188	Unhealthy snacking	“Eating high-calorie snacks”	“To avoid high-calorie snacks”	SRHI (.89)	.90	-	.50^a^	.26	.89			
					SRBAI (.84)		-	.42^b^	.19	.35			
					Non-SRBAI (.81)		-	.50^a^	.27	.95			
Dataset 4: *New dataset*	204	Alcohol consumption with the evening meal	“Drinking an alcoholic drink with my evening meal”	“To drink an alcoholic drink with my evening meal”	SRHI (.89)	.95	-	.80 ^a^	.68	.14			
					SRBAI (.84)		-	.75 ^b^	.64	.02	.56***	.46***	.35***
					Non-SRBAI (.81)		-	.80 ^a^	.68	.18			

*p < .05, ** *p* < .01, *** *p* < .001. Further details and analyses of all datasets are available on request from the first author.

**†** Ns are reduced for correlations with RFM in Datasets 1 (*N* = 102) and 2 (*N* = 99) due to missing RFM data.

**††** Differing superscript letters in ‘habit-behaviour’ column indicate differences in the magnitude of habit-behaviour correlations at *p* < .05 (see [37]). Correlations with the transport-specific RFM were only available in Datasets 1 and 2. All correlations significant at *p* < .01.

**†††** All regression models were significant at *p* < .001.

**††††** ‘Moderation effect’ refers to the predictive impact of a means-centred habit x intention interaction term on behaviour, controlling for habit and intention as independent predictors. Simple slope coefficients are provided for significant moderation effects only (*p* < .05).

#### Moderation tests

In Datasets 1 and 2, the SRBAI, SRHI, and ‘non-SRBAI’ indices each moderated the intention-behaviour relationship in line with theoretical predictions (maximum *p* = .04), with intention-behaviour relations strongest at low habit, and weakening as habit strength increased. No moderation was found using any index in Dataset 3 (minimum *p* = .35). In Dataset 4, the SRHI (*β* = −.10, *p* = .14) and ‘non-SRBAI’ (*β* = −.09, *p* = .18) did not moderate the intention-behaviour relationship, but the SRBAI did (*β* = −.16, *p* = .02), such that the impact of intention on action weakened as habit strength increased. Thus, both Hypotheses 2a and 2b received mixed support. In Datasets 3 and 4, the SRBAI-based model explained less variance in behaviour (R^2^ = .19 and .64, respectively) than did the SRHI (R^2^ = .26 and .68) or SRBAI (R^2^ = .26 and .68), likely due to omission of items relating more to behaviour frequency than automaticity.

## Discussion

We have argued that the impact of habit on behavioural repetition can be more parsimoniously captured by a subset of automaticity items from the Self-Report Habit Index (SRHI). It has been suggested elsewhere that automaticity is the ‘active ingredient’ of habit [15,24], and so we extracted from the SRHI a four-item automaticity subscale (the ‘Self-Report Behavioural Automaticity Index’; SRBAI). We assessed the utility of the SRBAI in a re-analysis of data from all available previous SRHI applications, and primary analyses of four energy-balance related behaviours (EBRBs): inactive (car) commuting, active (bicycle) commuting, unhealthy snacking, and alcohol consumption. The SRBAI was found to meet criteria that have been taken to reflect the adequacy of the SRHI for detecting health habits: reliability, convergent validity, and predictive validity. The SRBAI was reliable, and correlated strongly with the SRHI, and, in travel mode choice applications, the Response-Frequency Habit Measure (RFM). The SRBAI was also sensitive to effects predicted by theory [5], correlating with behaviour frequency, and typically detecting the moderating impact of habit on the intention-behaviour relation. In an application to alcohol consumption, the SRBAI was more sensitive to hypothesised moderation than was the SRHI, or the eight-item subset excluded from the SRBAI (the ‘non-SRBAI’). On balance, the SRBAI operated in close concordance with the SRHI in detecting habit-behaviour relationships, despite the removal of eight items.

The SRHI is the most commonly used measure of nutrition and activity habits [4], but its popularity does not necessarily reflect parsimony. Similar sensitivity of the SRBAI and SRHI to hypothesised habit effects suggests that our measure is more succinct and practical than the SRHI. This makes the SRBAI well suited to study of EBRBs, in which multiple determinants, habits and behaviours contribute to a positive energy balance. While SRHI subscales have been used previously [3,16-18], our work is the first to have systematically extracted core items using robust content validity techniques. Adoption of the SRBAI as the standard SRHI shortform, at least in determinant studies and studies of habit formation and disruption, would aid future research by offering an SRHI subscale that best captures characteristic habit-behaviour effects [15,24,25]. Use of this measure would also ensure homogeneity of measurement for future research syntheses.

There are also conceptual advantages to our subscale. It assesses one characteristic of behaviour patterns, distinguishing automaticity from behavioural frequency and behavioural identification. It has been proposed that automaticity is the ‘active ingredient’ of habit, and that the inclusion of non-automaticity indicators in the SRHI may confound detection of true habit-behaviour effects [4,15,24]. Social cognition theories posit that habit will moderate the intention-behaviour relation, so that where habit strengthens, the intention-behaviour link is attenuated [5]. Across all datasets, the SRBAI was equally sensitive to the SRHI in detecting hypothesised moderation in eight of eleven tests. One test showed the SRHI to detect moderation where the SRBAI did not, and in another test, the SRBAI detected moderation to which neither the SRHI nor the ‘non-SRBAI’ was sensitive. In a further test, a tendency towards moderation was observed using the SRBAI, though this ‘effect’ was underpinned by *stronger* intention-behaviour relations as habit strength increased [44]. Notably however, in this test the SRHI tended towards moderation in the same direction as did the SRBAI. Although empirically well supported elsewhere [4], the validity of moderation as a criterion for a habit index was challenged by the failure of either the SRHI or SRBAI to reliably detect moderation in three datasets, and the unexpected strengthening of intention-behaviour relations in another dataset. These findings may perhaps reflect measurement artefacts arising from consistency biases or limited range in habit or intention measures. Further work is needed to explore the conditions in which the hypothesised moderating impact of habit on the intention-behaviour relation best holds when assessed by self-report data.

The SRHI, and a subscale of items excluded from the SRBAI, predicted more variance in behaviour than did the SRBAI in snacking and alcohol consumption applications. It might therefore be argued that while we have added parsimony to the SRHI, by doing so we have compromised its predictive validity. We do not however believe that stronger habit-behaviour correlations necessarily reflect superior predictive validity of the SRHI: if the impact of habit on action can be solely attributed to automaticity [15,24] then the additional variance accounted for by the SRHI and ‘non-SRBAI’ scales may not be reliably attributable to habit. Previous research has shown that self-identity, which is also measured by the SRHI, correlates with behavioural frequency but, unlike habit, does not predict behaviour directly [14]. Concerns have also been raised about the validity of including frequency indicators in the SRHI when estimating habit-behaviour relationships, because behaviour frequency can capture both automatic (habitual) and reflective (non-habitual) influences on action [26]. We suggest that the eight items excluded from the SRBAI, which likely capture identity-relevance, behavioural frequency, and weaker automaticity indicators, may therefore correlate with behaviour independently of automaticity, so inflating true relationships between automatic cue-responding and behaviour frequency [4]. Our preliminary content validation procedure identified seven potential automaticity indicators however, and future work might test this explanation by assessing whether a seven-item automaticity index improves on the predictive validity of the SRBAI. Any such gains in predictive validity would however need to be sufficiently sized to justify foregoing the parsimony benefits afforded by the four-item SRBAI.

A measure of a psychological construct can be considered useful in at least two respects: first, detection of the corollaries of the construct, and second, demarcation of the construct. Habits are distinct from other forms of automaticity in that they are learned through repetition in stable contexts, and are ongoing, having previously been enacted and remaining likely to be enacted in future encounters with associated environmental cues [2,3]. Our results suggest that the SRBAI can adequately and concisely detect the effects of habit on behaviour, but it is unlikely to distinguish habit-related automaticity from other forms of automaticity, such as behaviour prompted by implementation intentions (i.e. one-off, pre-planned and highly specific cue-responses [46]), or ideomotor or primed behaviours [47,48]. Items relating to repetition history may be needed to distinguish habit from non-habit-related automaticity, and for these reasons, we term our measure an index of automaticity, rather than a measure of habit per se. The SRHI is however most commonly applied to behaviour prediction and habit formation studies, in which such a distinction is not of interest, and in these research contexts the SRBAI offers a more practical and parsimonious alternative to the SRHI.

Criticisms of the SRHI have been raised which are not addressed by our subscale. For example, the validity of self-reports on action which may proceed outside of awareness has been questioned [15,49]. The utility of the SRHI suggests that these concerns may be overstated because people are commonly *aware when reflecting retrospectively* on their behaviour that they were *not consciously monitoring the behaviour when it was enacted.* Validation of both the SRHI and SRBAI against lab-based measures of automated action is needed to support this assertion [50]. Commentators have also suggested that the SRHI is limited because it typically omits cues to habits [15,20]. Our alcohol consumption application demonstrated that the measure could be worded to include a contextual component (i.e. ‘drinking alcohol *with my evening meal*’), but the idiosyncratic nature of habit cues, and potential lack of awareness of the specific cues to habitual action, remains problematic for the validity of the SRHI and SRBAI. SRHI applications have also been criticised for relying on correlational and often cross-sectional population-level survey data [15], and we recognise the limitations of using such data to understand person- and context-specific cue-response associations. Nonetheless, the SRHI has come to be accepted as an adequate measure of habit on the basis of analyses of such data. Our data thus indicate that the SRBAI meets the same criteria by which the SRHI has previously been judged, as applied in the research contexts in which the SRHI has been most frequently used.

## Conclusion

We have argued that the impact of habit on behaviour can be measured more parsimoniously by using clearly-defined automaticity items from the Self-Report Habit Index. Our four-item SRHI subscale is more succinct and easier to administer than extant measures. This measure, the Self-Report Behavioural Automaticity Index, correlates highly with existing measures, and appears sensitive to effects that characterise habits. It offers practical benefits for detection of EBRB habits, and we recommend its use in behaviour prediction studies, and studies that track habit formation or disruption.

## Abbreviations

EBRB: Energy-balance related behaviour; MMR: Moderated multiple regression; RFM: Response-frequency habit measure; SRHI: Self-report habit index; SRBAI: Self-report behavioural automaticity index.

## Competing interests

The authors declare that they have no competing interests.

## Authors’ contributions

BG and CA formulated the initial research ideas and generated specific hypotheses in collaboration with PL and GJdB. BG collected and analysed all data, and drafted the manuscript. CA oversaw design, collection and reporting of work on Datasets 1 and 2 as supervisor of BG’s doctoral research. GJdB and PL assisted with selection and coding of secondary data. GJdB assisted with analysis of secondary data. All authors contributed to redrafts of the manuscript, and read and approved the final manuscript.

## Supplementary Material

Additional file 1 **Figure S1.** Results of systematic search strategy and screening procedure. Click here for file

Additional file 2 **Table S1.** Secondary datasets: Study characteristics, reliabilities, and habit-behaviour and SRHI-SRBAI correlations. Click here for file

Additional file 3 **Table S2a.** Primary datasets: Descriptives and intercorrelations (Datasets 1 and 2). Click here for file

Additional file 4 **Table S2b.** Primary datasets: Descriptives and intercorrelations (Datasets 3 and 4). Click here for file

Additional file 5 **References for Supplementary Material **[51-79]. Click here for file
