# Supplementary material for: Towards parsimony in habit measurement: Testing the convergent and predictive validity of an automaticity subscale of the Self-Report Habit Index
Source: Int J Behav Nutr Phys Act. 2012 Aug 30;9:102. doi: 10.1186/1479-5868-9-102 (PMC3552971; doi:10.1186/1479-5868-9-102)
Supplement: Additional file 1 — Figure S1. Results of systematic search strategy and screening procedure. [file 1479-5868-9-102-S1.doc]

**Supplementary Figure 1:** Results of systematic search strategy and screening procedure

**Identification**

Papers identified through database searching (k = 312)

Additional papers identified through other sources (k = 4)

**Included**

**Eligibility**

*Full-text papers excluded
(k = 85)*

*Reasons*

- No primary data (k = 28)

- No habit measure (n = 18)

- Non-SRHI habit measure (n = 16)

- <12 SRHI items (n = 18)

- Mental habit only (n = 3)

- No data for any of 3 analyses (k = 2)

Titles/abstracts screened
(k = 139)

*Papers excluded*

*(k = 7)*

*Reasons*

- Non-English (k = 1)

- Not published as full-text (k = 1)

- No primary data (k = 5)

Full-text papers assessed for eligibility (k = 132)

Potentially eligible papers

(k = 47 [49 relevant datasets])

Papers after duplicates removed
(k = 135)

Included in **reliability analysis**

(k = 39 [34 datasets, 45 tests])

Entered into **correlation analysis**

(k = 26 [24 datasets, 28 tests])

Included in **moderation analysis**

(k = 7 [5 datasets, 7 tests])

**Screening**

*Papers excluded
(k = 8 [15 datasets])*

*Reasons*

- Insufficient data from authors (k = 7 [13 datasets])

- Paper omitted from secondary analysis because studies included in primary analyses in this paper (k = 1 [2 datasets])
