# Supplementary material for: Towards parsimony in habit measurement: Testing the convergent and predictive validity of an automaticity subscale of the Self-Report Habit Index
Source: Int J Behav Nutr Phys Act. 2012 Aug 30;9:102. doi: 10.1186/1479-5868-9-102 (PMC3552971; doi:10.1186/1479-5868-9-102)
Supplement: Additional file 2 — Table S1. Secondary datasets: Study characteristics, reliabilities, and habit-behaviour and SRHI-SRBAI correlations. [file 1479-5868-9-102-S2.doc]

**Supplementary Table 1.** *Secondary datasets:* Study characteristics, reliabilities, and habit-behaviour and SRHI-SRBAI correlations

| **Reference / Dataset** | **N** | **Behaviour** | **Habit** | **Reliability α** | | **Correlations** | | | |
| --- | --- | --- | --- | --- | --- | --- | --- | --- | --- |
|  |  |  |  |  |  | **SRHI-SRBAI** | **Habit-behaviour** | | |
|  |  |  |  | ***SRHI*** | ***SRBAI*** |  | ***SRHI*** | ***SRBAI*** | ***Z*** |
| Adriaanse, de Ridder & Evers ([1], Study 1) | 149 | Unhealthy snacking  (SR) | “Eating unhealthy snacks” | **.94** | **.86** | **.88** | **.43** | **.32** | 2.94** |
| Adriaanse, de Ridder & Evers ([1], Study 2) | 182 | Unhealthy snacking  (SR) | “Eating unhealthy snacks” | **.90** | **.72** | **.84** | **.29** | **.25** | 0.99 |
| Adriaanse, Oettingen et al ([2], Study 1) | 51 | *N/A* | “Eating unhealthy snacks” | **.90** | **.84** | **.82** | *N/A* | *N/A* | *N/A* |
| Adriaanse, Oettingen et al ([2], Study 2) | 59 | *N/A* | (Miscellaneous) | **.89** | **.86** | **.88** | *N/A* | *N/A* | *N/A* |
| Adriaanse, van Oosten et al ([3], Study 4) | 61 | *N/A* | “Eating unhealthy snacks” | **.94** | **.90** | **.90** | *N/A* | *N/A* | *N/A* |
| Bolman, Arwert & Vollink [4] | 130 | Prophylactic asthma medication adherence (SR) | “Inhaling prophylactic asthma medication” | **.93** | **.86** | **.92** | **.61** | **.54** | 2.44** |
| Conner et al ([5], Study 1) | 120 | Sweet consumption  (SR) | “In general, eating sweets and chocolate” | **.89** | **.86** | **.79** | **.39** | **.33** | 1.02 |
| Conner et al ([5], Study 2)‡ | 97 | Choice of chocolate rather than fruit  (O) | “In general, eating fruit” | **.86** | **.75** | **.86** | **-.16**‡‡ | **-.15**‡‡ | 0.19 |
| De Bruijn [6] | 330 | *N/A* | “Exercising on at least 3 days a week for at least 20mins per bout” | **.97** | **.96** | **.94** | *N/A* | *N/A* | *N/A* |
| De Bruijn [7] */ De Bruijn & Gardner [8]/ De Bruijn & Rhodes [9]* † | 538 | Fruit consumption  (SR) | “Eating at least 2 pieces of fruit per day” | **.95** | **.91** | **.94** | **.53** | **.46** | 5.41*** |
| *De Bruijn [7] /* De Bruijn & Gardner [8]/ *De Bruijn & Rhodes [9]* † | 538 | Bicycle use  (SR) | “Using a bicycle for transportation purposes” | **.97** | **.94** | **.95** | **.39** | **.34** | 3.94*** |
| *De Bruijn [7] / De Bruijn & Gardner [8]/* De Bruijn & Rhodes [9] † | 538 | Physical activity  (SR) | “Exercising on at least 3 days a week for at least 20mins per day” | **.97** | **.94** | **.95** | **.35** | **.31** | 3.10*** |
| De Bruijn, Kremers, De Vet et al [10] */ Brug et al [11]* | 876 | Fruit consumption  (SR) | “Eating two servings of fruit per day” | **.97** | **.93** | **.95** | **.57** | **.52** | 5.61*** |
| De Bruijn, Kroeze et al [12] | 748 | Fat consumption  (SR) | “Watching the amount of fat in my diet” | **.94** | **.85** | **.92** | **-.26**‡‡ | **-.23**‡‡ | 2.11* |
| De Bruijn, Kremers, Singh et al [13] | 317 | Bicycle use  (SR) | Using a bicycle as a means of transportation | **.98** | **.95** | **.97** | **.57** | **.51** | 5.18*** |
| De Bruijn & van den Putte [14] †† | 312 | TV-viewing  (SR) | “Watching television” | **.89** | **.87** | **.89** | **.44** | **.39** | 2.07* |
| De Bruijn & van den Putte [14] †† | 312 | Sugar-sweetened soft drink consumption  (SR) | “Drinking  sugar-sweetened soft drinks” | **.95** | **.91** | **.93** | **.49** | **.43** | 3.19*** |
| Eriksson, Nordlund & Garvill [15] | 69 | Car use  (SR) | “Using a car” | **.94** | **.93** | **.92** | **.29** | **.26** | 0.64 |
| Fischer, Frewer & Nauta [16] / Fischer & Frewer [17] | 1029 | *N/A* | “Preparing the daily hot meal” | **.89** | **.81** | **.82** | *N/A* | *N/A* | *N/A* |
| Hinsz, Nickell & Park [18] | 162 | Safe preparation of turkey products  (SR) | “(Doing) all that is needed to produce clean and uncontaminated turkey products” | **.78** | **.68** | **.83** | **.48** | **.41** | 1.71* |
| Jansson, Marell & Nordlund [19,20] | 1672 | Non-car travel (SR) | “Using the car” | **.95** | **.91** | **.93** | **-.55**‡‡ | **-.51**‡‡ | 5.19*** |
| Jurg et al [21] / Kremers & Brug ([22], Study 1) / Kremers, Dijkman et al [23] | 502 | Physical activity  (SR) | “Exercise” | **.84** | **.70** | **.80** | **.33** | **.18** | 5.50*** |
| Klöckner & Oppedal [24] | 664 | *N/A* | “Recycling”? | **.96** | **.93** | **.95** | *N/A* | *N/A* | *N/A* |
| Kovač & Rise ([25], Study 2) / Kovač, Rise & Moan [26] | 925 | Cigarette consumption (SR) | “Smoking” | **.89** | **.83** | **.92** | **.57** | **.52** | 4.57*** |
| Kremers & Brug ([22], Study 2) / Kremers, van der Horst & Brug [27] */ van der Horst et al [28]* ††† | 357 | TV or computer viewing (SR) | “Watching TV or using the computer” | **.90** | **.86** | **.85** | **.50** | **.37** | 5.01*** |
| *Kremers & Brug ([22], Study 2) /* Kremers, van der Horst & Brug [27] / van der Horst et al [28] ††† | 357 | Sugar-sweetened soft drink consumption  (SR) | “Drinking sugar-sweetened beverages” | **.89** | **.83** | **.85** | **.48** | **.38** | 3.84*** |
| Lally, Chipperfield & Wardle [29] | 98 | *N/A* | (Miscellaneous) | **.97** | **.95** | **.97** | *N/A* | *N/A* | *N/A* |
| Lemieux & Godin [30] | 130 | Active commuting  (SR) | “Using active commuting” | **.96** | **.97** | **.97** | **.60** | **.61** | -0.58 |
| Lucas et al ([31], Sample 1) ††††, ‡‡‡ | 152 | *N/A* | “Drinking alcohol” | **(.93)** | **.90** | **.84** | *N/A* | *N/A* | *N/A* |
| Lucas et al ([31], Sample 1) ††††, ‡‡‡ | 152 | *N/A* | “Smoking” | **(.97)** | **.93** | **.94** | *N/A* | *N/A* | *N/A* |
| Lucas et al ([31], Sample 1) ††††, ‡‡‡ | 152 | *N/A* | “Eating healthy” | **(.97)** | **.96** | **.96** | *N/A* | *N/A* | *N/A* |
| Lucas et al ([31], Sample 1) ††††, ‡‡‡ | 152 | *N/A* | “Exercising”? | **(.96)** | **.82** | **.93** | *N/A* | *N/A* | *N/A* |
| Lucas et al ([31], Sample 2) †††††, ‡‡‡ | 274 | *N/A* | “Drinking alcohol” | **(.93)** | **.88** | **.88** | *N/A* | *N/A* | *N/A* |
| Lucas et al ([31], Sample 2) †††††, ‡‡‡ | 274 | *N/A* | “Smoking” | **(.97)** | **.97** | **.97** | *N/A* | *N/A* | *N/A* |
| Lucas et al ([31], Sample 2) †††††, ‡‡‡ | 274 | *N/A* | “Eating healthy” | **(.97)** | **.95** | **.96** | *N/A* | *N/A* | *N/A* |
| Lucas et al ([31], Sample 2) †††††, ‡‡‡ | 274 | *N/A* | “Exercising”? | **(.96)** | **.93** | **.95** | *N/A* | *N/A* | *N/A* |
| Norman [32] | 109 | Binge-drinking  (SR) | “Binge-drinking” | **.94** | **.90** | **.93** | **.60** | **.54** | 2.03* |
| Norman & Cooper [33] | 66 | Breast self-examination (SR) | “Breast self-examination” | **.95** | **.94** | **.93** | **.28** | **.22** | 1.32 |
| Pearson et al [34] | 49 | *N/A* | “Eating fruit and vegetables” | **.92** | **.89** | **.93** | *N/A* | *N/A* | *N/A* |
| Rhodes, de Bruijn & Matheson [35] | 153 | Physical activity  (SR) | “Engaging in active sports and/or vigorous physical activities during my leisure time” | **.97** | **.93** | **.95** | **.55** | **.50** | 2.53** |
| Tam, Bagozzi & Spanjol [36] | 129 | Healthy snack consumption (SR) | “Unhealthy snacking” | **.92** | **.85** | **.96** | **-.11**‡‡ | **-.14**‡‡ | -1.20 |
| Verplanken ([37], Study 1) | 110 | Unhealthy snacking (SR) | “Eating snack food” | **.87** | **.75** | **.83** | **.51** | **.37** | 2.80** |
| Verplanken & Melkevik [38] | 106 | Physical activity (SR) | “Exercising” | **.93** | **.83** | **.94** | **.51** | **.44** | 2.34* |
| Weijzen, de Graaf & Dijksterhuis [39] | 536 | Choice of healthy vs unhealthy snack  (O) | “Choosing a healthy snack” | **.92** | **.80** | **.88** | **.27** | **.17** | 4.84*** |

* p<.05, **p<.01, ***p<.001. P values are one-tailed. Z refers to the difference between correlated correlation coefficients, calculated using Meng et al’s guidelines [40]. SR = Self-report. O = Objective. PA = Physical activity. Behaviours were coded so that higher scores indicate greater engagement in behaviour. References marked with crosses (†) report multiple relevant habit-behaviour correlations from a single dataset; the number of crosses assigned is unique to each such dataset. Italicised references indicate papers drawn from a single dataset which are of lesser relevance to the corresponding row. Data may differ from that reported in published papers because we prioritised data provided to us by authors. ‡ Correlations between habit and directly-opposed behaviours were reversed for meta-analysis purposes. ‡‡ One dataset [5] used two habit measures (fruit consumption, chocolate consumption) that each correlated with the same behaviour measure; to avoid over-representation, we excluded the ‘chocolate’ habit measure based on its weaker reliability (chocolate: SRHI α = .86, SRBAI α =.75; fruit: SRHI α = .92, SRBAI α = .91). ‡‡‡ In one paper, which featured multiple habit measures, the total sample was discerned into two sub-samples [31]. The corresponding author of this paper provided SRBAI reliability and SRHI-SRBAI correlation coefficients, but not SRHI reliability coefficients, for each sub-sample. Total-sample SRHI alphas (in parentheses) have been substituted for sub-sample SRHI alphas here.
