# Supplementary material for: Towards parsimony in habit measurement: Testing the convergent and predictive validity of an automaticity subscale of the Self-Report Habit Index
Source: Int J Behav Nutr Phys Act. 2012 Aug 30;9:102. doi: 10.1186/1479-5868-9-102 (PMC3552971; doi:10.1186/1479-5868-9-102)
Supplement: Additional file 3 — Table S2a. Primary datasets: Descriptives and intercorrelations (Datasets 1 and 2). [file 1479-5868-9-102-S3.doc]

**Supplementary Table 2a.** *Primary datasets:* Descriptives and intercorrelations (Datasets 1 and 2)

| *Dataset 2 (bicycle commuting)* | *Dataset 1 (car commuting)* | | | | | | | | | | | |  |
| --- | --- | --- | --- | --- | --- | --- | --- | --- | --- | --- | --- | --- | --- |
|  |  | *1.* | *2.* | *3.* | *4.* | *5.* | *6.* | *N* | *Range* | *Mean* | *SD* | |  |
|  | 1. Behaviour (T2) | - | .86 | .82 | .81 | .45 | .83 | 105 | 1-100 | 77.79 | 36.03 | |  |
|  | 2. SRHI | .86 | - | .94 | .98 | .52 | .90 | 105 | 1-7 | 4.59 | 1.81 | |  |
|  | 3. SRBAI | .86 | .97 | - | .86 | .52 | .80 | 105 | 1-7 | 4.49 | 2.11 | |  |
|  | 4. ‘Non-SRBAI’ | .84 | .99 | .92 | - | .49 | .90 | 105 | 1-7 | 4.64 | 1.75 | |  |
|  | 5. RFM | .62 | .67 | .65 | .67 | - | .45 | 102 | 1-10 | 5.42 | 2.77 | |  |
|  | 6. Intention | .80 | .86 | .84 | .85 | .65 | - | 105 | 1-7 | 5.05 | 2.33 | |  |
|  | *N* | 102 | 102 | 102 | 102 | 99 | 102 |  |  |  |  | |  |
|  | *Range* | 1-100 | 1-7 | 1-7 | 1-7 | 1-10 | 1-7 |  |  |  |  |  | |
|  | *Mean* | 78.17 | 5.18 | 5.34 | 5.09 | 6.11 | 5.93 |  |  |  |  |  | |
|  | *SD* | 36.56 | 1.67 | 1.89 | 1.60 | 2.49 | 1.81 |  |  |  |  |  | |

Values above the diagonal refer to Dataset 1, and values below the diagonal to Dataset 2. All *p*s<.001.
