# Supplementary material for: Towards parsimony in habit measurement: Testing the convergent and predictive validity of an automaticity subscale of the Self-Report Habit Index
Source: Int J Behav Nutr Phys Act. 2012 Aug 30;9:102. doi: 10.1186/1479-5868-9-102 (PMC3552971; doi:10.1186/1479-5868-9-102)
Supplement: Additional file 4 — Table S2b. Primary datasets: Descriptives and intercorrelations (Datasets 3 and 4). [file 1479-5868-9-102-S4.doc]

***Supplementary Table 2b.*** *Primary datasets:* *Descriptives and intercorrelations (Datasets 3 and 4)*

| *Dataset 4 (Alcohol with evening meal; N = 204)* |  | *Dataset 3 (unhealthy snacking; N = 188)* | | | | | | | | | | | |
| --- | --- | --- | --- | --- | --- | --- | --- | --- | --- | --- | --- | --- | --- |
|  |  | | *1.* | *2.* | *3.* | *4.* | *5.* | *Range* | *Mean* | *SD* | |  | |
|  | 1. Behaviour† | | - | .50 | .42 | .50 | -.28 | 1-35 | 10.32‡ | 2.89 | |  | |
|  | 2. SRHI | | .80 | - | .90 | .96 | -.36 | 1-7 | 3.50 | 1.19 | |  | |
|  | 3. SRBAI | | .75 | .95 | - | .73 | -.39 | 1-7 | 3.39 | 1.55 | |  | |
|  | 4. ‘Non-SRBAI’ | | .80 | .99 | .90 | - | -.29 | 1-7 | 3.55 | 1.13 | |  | |
|  | 5. Intention | | .74 | .79 | .75 | .78 | - | 1-7 | 4.06 | 1.81 | |  | |
|  | *Range* | | 1-100 | 1-7 | 1-7 | 1-7 | 1-7 |  |  |  |  | |  |
|  | *Mean* | | 27.57‡‡ | 2.14 | 1.90 | 2.26 | 2.11 |  |  |  |  | |  |
|  | *SD* | | 28.05‡‡ | 1.41 | 1.35 | 1.48 | 1.80 |  |  |  |  | |  |

NB: Values above the diagonal refer to Dataset 3, and values below the diagonal to Dataset 4. All *p*s<.01. † Dataset 3 used a prospective design and Dataset 4 used a cross-sectional design, and so behaviour measures refer to past behaviour in Dataset 3 and follow-up behaviour (one week post-baseline) in Dataset 4. ‡ Dataset 3 behaviour values refer to the summed frequency with which each of five snacks were eaten over a one-week period. ‡‡ Dataset 4 behaviour values refer to percentage of meals with which alcohol was consumed.
