# Supplementary material for: Towards parsimony in habit measurement: Testing the convergent and predictive validity of an automaticity subscale of the Self-Report Habit Index
Source: Int J Behav Nutr Phys Act. 2012 Aug 30;9:102. doi: 10.1186/1479-5868-9-102 (PMC3552971; doi:10.1186/1479-5868-9-102)
Supplement: Additional file 5 — References for Supplementary Material [51-79]. [file 1479-5868-9-102-S5.doc]

**References for Supplementary Material**

** References marked with an asterisk were at least partly based on data included in the review of secondary datasets.*

(1) * Adriaanse MA, de Ridder DTD, Evers C. Emotional eating: Eating when emotional or emotional about eating? *Psychol Health* 2011;26:23-39.

(2) * Adriaanse MA, Oettingen B, Gollwitzer PM, Hennes EP, de Ridder DTD, de Wit JBF. When planning is not enough: Fighting unhealthy snacking habits by mental contrasting with implementation intentions (MCII). *Euro J Soc Psychol* 2010;40:1277-1293.

(3) * Adriaanse MA, van Oosten JMF, de Ridder DTD, de Wit JBF, Evers C. Planning what not to eat: Ironic effects of implementation intentions negating unhealthy habits. *Pers Soc Psychol Bull* 2011;37:69-81.

(4) * Bolman C, Arwert TG, Vollink T. Adherence to prophylactic asthma medication: Habit strength and cognitions. *Heart Lung* 2011;40:63-75.

(5) * Conner MT, Perugini M, O'Gorman R, Ayres K, Prestwich A. Relations between implicit and explicit measures of attitudes and measures of behavior: Evidence of moderation by individual difference variables. *Pers Soc Psychol Bull* 2007;33:1727-1740.

(6) * de Bruijn GJ. Exercise habit strength, planning and the theory of planned behaviour: An action control approach. *Psychol Sport Exerc* 2011;12:106-114.

(7) * de Bruijn GJ. Understanding college students' fruit consumption. Integrating habit strength in the theory of planned behaviour. *Appetite* 2010;54:16-22.

(8) * de Bruijn GJ, Gardner B. Active commuting and habit strength: an interactive and discriminant analyses approach. *Am J Health Promot* 2011;25:e27-e36.

(9) * de Bruijn GJ, Rhodes RE. Exploring exercise behavior, intention and habit strength relationships. *Scand J Med Sci Sport* 2011;21:482-491.

(10) * de Bruijn GJ, Kremers SPJ, De Vet E, De Nooijer J, Van Mechelen W, Brug J. Does habit strength moderate the intention-behaviour relationship in the Theory of Planned Behaviour? The case of fruit consumption. *Psychol Health* 2007;22:899-916.

(11) * Brug J, de Vet E, de Nooijer J, Verplanken B. Predicting Fruit Consumption: Cognitions, Intention, and Habits. *J Nutr Educ Behav* 2006;Vol.38:73-81.

(12) * de Bruijn GJ, Kroeze W, Oenema A, Brug J. Saturated fat consumption and the Theory of Planned Behaviour: Exploring additive and interactive effects of habit strength. *Appetite* 2008;51:318-323.

(13) * de Bruijn GJ, Kremers SPJ, Singh A, van den Putte B, Van Mechelen W. Adult Active Transportation: Adding Habit Strength to the Theory of Planned Behavior. *Am J Prev Med* 2009;36:189-194.

(14) * de Bruijn GJ, van den Putte B. Adolescent soft drink consumption, television viewing and habit strength. Investigating clustering effects in the Theory of Planned Behaviour. *Appetite* 2009;53:66-75.

(15) * Eriksson L, Garvill J, Nordlund AM. Interrupting habitual car use: The importance of car habit strength and moral motivation for personal car use reduction. [References]. *Transport Research F – Traf.* 2008;11:10-23.

(16) * Fischer ARH, Frewer LJ, Nauta MJ. Toward improving food safety in the domestic environment: A multi-item Rasch scale for the measurement of the safety efficacy of domestic food-handling practices. *Risk Anal* 2006;26:1323-1338.

(17) * Fischer ARH, Frewer LJ. Food-safety practices in the domestic kitchen: Demographic, personality, and experiential determinants. *J Appl Soc Psychol* 2008;38:2859-2884.

(18) * Hinsz VB, Nickell GS, Park ES. The role of work habits in the motivation of food safety behaviors. *J Exp Psychol -Appl* 2007;13:105-114.

(19) * Jansson J, Marell A, Nordlund A. Elucidating green consumers: A cluster analytic approach on proenvironmental purchase and curtailment behaviors. *J Euromarketing* 2009;18:245-267.

(20) * Jansson J, Marell A, Nordlund A. Green consumer behavior: Determinants of curtailment and eco-innovation adoption. *J Consum Mark* 2010; 27:358-370.

(21) * Jurg ME, Kremers SPJ, Candel MJJM, van der Wal MF, de Meij JSB. A controlled trial of a school-based environmental intervention to improve physical activity in Dutch children: JUMP-in, kids in motion. *Health Prom Int* 2006; 21:320-330.

(22) * Kremers SPJ, Brug J. Habit strength of physical activity and sedentary behavior among children and adolescents. *Pediatr Exerc Sci* 2008;20:5-14.

(23) * Kremers SPJ, Dijkman MAM, De Meij JSB, Jurg ME, Brug J. Awareness and habit: Important factors in physical activity in children. *Health Educ* 2008;108:475-488.

(24) * Klöckner CA, Oppedal IO. General vs. domain specific recycling behaviour-Applying a multilevel comprehensive action determination model to recycling in Norwegian student homes. *Resour Conserv Recy* 2011;55:463-471.

(25) * Kovač VB, Rise J. The role of explicit cognition in addiction: Development of the mental representations scale. *Addict Res Theory* 2008;16:595-606.

(26) * Kovač VB, Rise J, Moan IS. From intentions to quit to the actual quitting process: The case of smoking behavior in light of the TPB. *J Appl Biobehav Res* 2009;14:181-197.

(27) * Kremers SPJ, Van der Horst K, Brug J. Adolescent screen-viewing behaviour is associated with consumption of sugar-sweetened beverages: The role of habit strength and perceived parental norms. *Appetite* 2007;48:345-350.

(28) * Van der Horst K, Kremers S, Ferreira I, Singh A, Oenema A, Brug J. Perceived parenting style and practices and the consumption of sugar-sweetened beverages by adolescents. *Health Educ Res* 2007;22:295-304.

(29) * Lally P, Chipperfield A, Wardle J. Healthy habits: Efficacy of simple advice on weight control based on a habit-formation model. *Int J Obes* 2008;32:700-707.

(30) * Lemieux M, Godin G. How well do cognitive and environmental variables predict active commuting? *Int J Behav Nutr Phy* 2009;6.

(31) * Lucas T, Alexander S, Firestone I, Lebreton JM. Just world beliefs, perceived stress, and health behavior: The impact of a procedurally just world. *Psychol Health* 2008;23:849-865.

(32) * Norman P. The theory of planned behavior and binge drinking among undergraduate students: Assessing the impact of habit strength. *Addict Behav* 2011;36:502-507.

(33) * Norman P, Cooper Y. The theory of planned behaviour and breast self-examination: Assessing the impact of past behaviour, context stability and habit strength. *Psychol Health* 2011;26:1156-1172.

(34) * Pearson N, Atkin AJ, Biddle SJH, Gorely T. A family-based intervention to increase fruit and vegetable consumption in adolescents: a pilot study. *Public Health Nutr* 2010;13:876-885.

(35) * Rhodes R, de Bruijn GJ, Matheson DH. Habit in the physical activity domain: Integration with intention temporal stability and action control. *J Sport Exerc Psychol* 2010; 32:84-98.

(36) * Tam L, Bagozzi RP, Spanjol J. When planning is not enough: The self-regulatory effect of implementation intentions on changing snacking habits. *Health Psychol* 2010; 29:284-292.

(37) * Verplanken B. Beyond frequency: Habit as mental construct. *Br J Soc Psychol* 2006;45:639-656.

(38) * Verplanken B, Melkevik O. Predicting habit: The case of physical exercise. *Psychol Sport Exerc* 2008;9:15-26.

(39) * Weijzen PLG, de Graaf C, Dijksterhuis GB. Predictors of the consistency between healthy snack choice intentions and actual behaviour. *Food Qual Prefer* 2009;20:110-119.

(40) Meng X-L, Rosenthal R, Rubin DB. Comparing correlated correlation coefficients. *Psychol Bull* 1992;111:172-175.
